# The Evolution of the Healthy Brain Initiative

**DOI:** 10.1093/geroni/igaa057.2542

**Published:** 2020-12-16

**Authors:** Lisa McGuire

**Affiliations:** Centers for Disease Control and Prevention, Atlanta, Georgia, United States

## Abstract

The Healthy Brain Initiative (HBI) seeks to advance public health awareness of and action on ADRD as a public health issue. The HBI Road Map Series, State and Local Public Health Partnerships to Address Dementia: The 2018–2023 Road Map (S&L RM) and Road Map for Indian Country (RMIC), provide the public health with concrete steps to respond to the growing burden of ADRD in communities, consistent with the aim of the Building Our Largest Dementia (BOLD) Infrastructure for Alzheimer’s Act (P.L. 115-406). This series of RMs for state, local, and tribal public health provide flexible menus of actions to address cognitive health, including ADRD, and support for dementia caregivers with population-based approaches. This session will describe how the initiative evolved over the past 15 years including policy and implementation success stories.

